# Kdm5/Lid Regulates Chromosome Architecture in Meiotic Prophase I Independently of Its Histone Demethylase Activity

**DOI:** 10.1371/journal.pgen.1006241

**Published:** 2016-08-05

**Authors:** Liudmila Zhaunova, Hiroyuki Ohkura, Manuel Breuer

**Affiliations:** Wellcome Trust Centre for Cell Biology, School of Biological Sciences, University of Edinburgh, Edinburgh, United Kingdom; Stowers Institute for Medical Research, UNITED STATES

## Abstract

During prophase of the first meiotic division (prophase I), chromatin dynamically reorganises to recombine and prepare for chromosome segregation. Histone modifying enzymes are major regulators of chromatin structure, but our knowledge of their roles in prophase I is still limited. Here we report on crucial roles of Kdm5/Lid, one of two histone demethylases in *Drosophila* that remove one of the trimethyl groups at Lys4 of Histone 3 (H3K4me3). In the absence of Kdm5/Lid, the synaptonemal complex was only partially formed and failed to be maintained along chromosome arms, while localisation of its components at centromeres was unaffected. Kdm5/Lid was also required for karyosome formation and homologous centromere pairing in prophase I. Although loss of Kdm5/Lid dramatically increased the level of H3K4me3 in oocytes, catalytically inactive Kdm5/Lid can rescue the above cytological defects. Therefore Kdm5/Lid controls chromatin architecture in meiotic prophase I oocytes independently of its demethylase activity.

## Author Summary

Accurate transmission of chromosomes carrying genetic materials from generation to generation is essential for life. Cell divisions that generate gametes, such as eggs and sperm, are critical, as chromosomes inherited from both parents recombine and are accurately sorted into gametes. Errors in these cell divisions often result in infertility, miscarriages or birth defects such as Down syndrome in humans. During these divisions, chromosomes undergo dramatic reorganisation but the molecular mechanisms are not well understood. Chromosome organisation is known to be regulated by various epigenetic marks, which are chemical marks on chromatin crucial for regulating gene expression. We found that an enzyme (Kdm5/Lid) that erases a mark linked to active gene expression regulates multiple aspects of meiotic chromatin organisation in oocytes, including stability of the recombination machinery. Unexpectedly, this function does not require its enzymatic activity. Our findings provide novel insights into how chromosomes are reorganised during reproduction and prompt re-evaluation of the role of this eraser enzyme.

## Introduction

Meiosis I differs from the second meiotic and mitotic divisions in that homologous chromosomes are segregated, not sister chromatids. Accurate chromosome segregation in meiosis I is preceded by dramatic changes in prophase chromatin organisation. Eventually, homologous chromosomes are mechanically held on the metaphase spindle via chromosome arm cohesion and the crossover-induced physical linkage of chiasmata [1]. This structural configuration is brought about by recombination, in the majority of organisms the driving force behind the alignment, or pairing of homologues, guiding their subsequent close association, or synapsis. Notably, in *C*. *elegans* and *Drosophila*, pairing and synapsis occur without double strand breaks (DSBs) and are thus recombination-independent [2,3].

A global feature of meiosis, however, is the instalment of a chromosome axis on each synapsed bivalent, which serves as a platform to enable the activities of the recombination complexes and, importantly, link the homologues along their entire length [4]. This connection is mediated by the synaptonemal complex (SC), a highly conserved proteinaceous structure with lateral and transverse elements. The full joining of SC elements along the homologues results in synapsis. Although the exact role of the SC in recombination is not fully dissected and may vary among organisms, it is thought to have a promoting effect on the maturation of homologue-exchange crossover events [5]. Thus, it establishes the prerequisite for chiasmata and therefore a suitable chromosome configuration for homologous segregation upon meiotic anaphase I. The chromosome axis is comprised of structural proteins like the cohesin complex, composed of the subunits SMC1 and SMC3 and two non-SMC subunits [6,7]. Enrichment of cohesins leads to shortening of sister chromatids along their longitudinal axis. Overall, these dramatic chromatin changes along with SC loading are key features of meiotic chromosome architecture. For the formation of crossovers, a role for chromatin structure rearrangements–nucleosome accessibility and histone modifications–has been described [8]. Histone trimethylation at H3 (H3K4me3) is located at DSB sites in hotspots in mammals and yeast, along with the H3K4 methyltransferase PRDM9 [9–12]. However, the exact mechanism on the chromosome axis for this higher order DNA structural feature is not known.

Another mechanism to alter nuclear chromatin organisation, to enable pairing, is the coupling of homologous centromeric regions in early meiosis [13]. *Drosophila* has adopted an extreme version of coupling, where after the breaking of somatic pairing, homologous and non-homologous centromeres are clustered even before the germline cells enter meiosis [14–17]. This clustering is maintained throughout oogenesis, post SC disassembly, when all homologous chromosomes have compacted into a spherical structure in the oocyte nucleus, the karyosome [18]. In the context of karyosome formation, a link has been shown between the underlying chromatin configuration via histone modifications and the global reorganisation of chromatin-associated protein complexes like the SC or condensin [19]. However, further regulators of chromatin architecture implicated in these meiotic reorganisations remained to be identified.

Here we show that the histone demethylase Kdm5/Lid controls chromatin architecture in prophase I oocytes, including the SC, pairing of homologous centromeres, and the karyosome. Interestingly, we found that although Kdm5/Lid is a major demethylase of H3K4me3, its demethylase activity is dispensable for prophase I chromatin architecture in *Drosophila* oocytes.

## Results

### Loss of the histone demethylase Kdm5/Lid results in a severe karyosome defect

**Fig 1:**
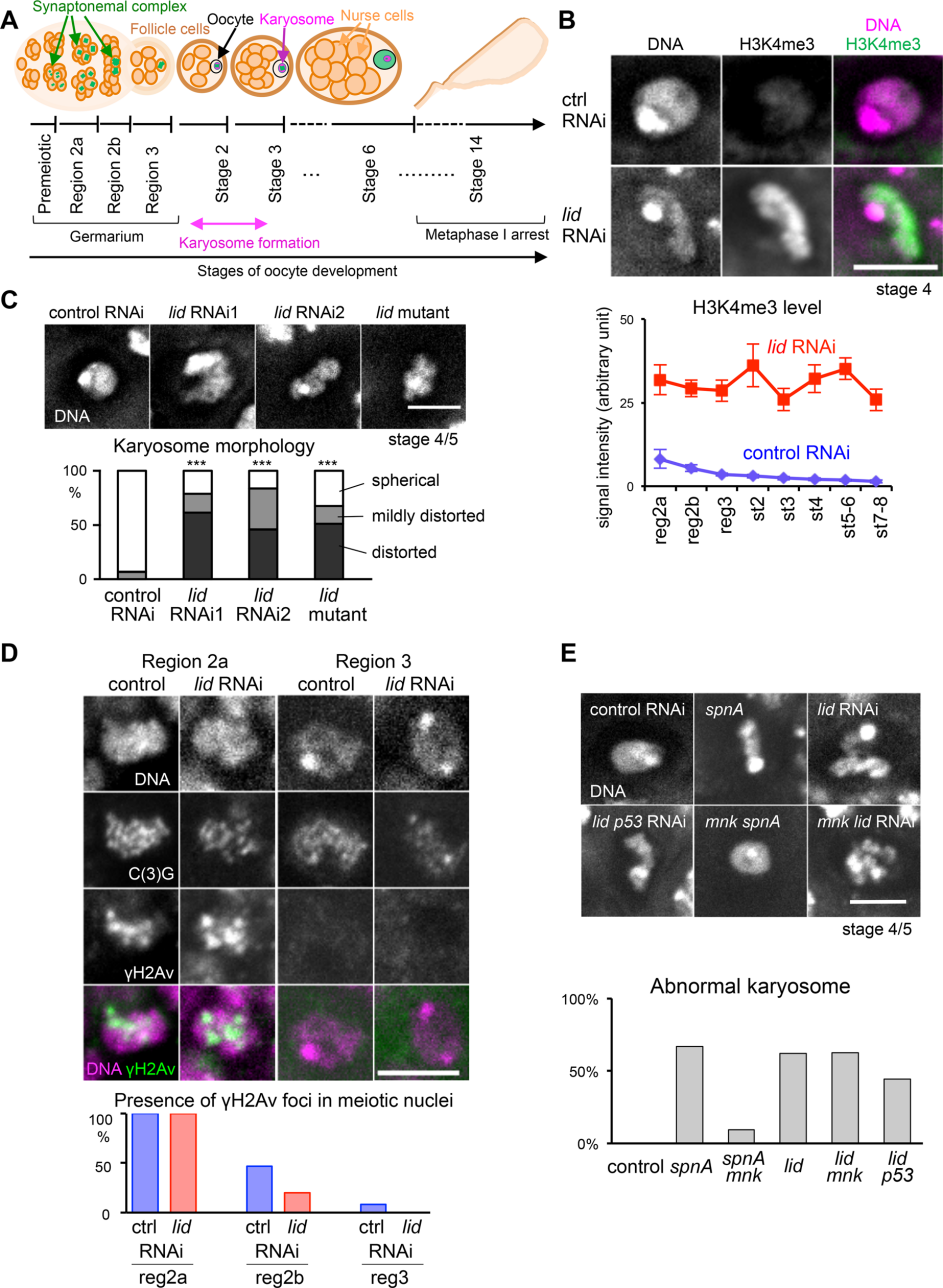
Loss of Kdm5/Lid results in high H3K4me3 and abnormal karyosomes independently from the meiotic recombination checkpoint. (A) Schematic representation of *Drosophila* oogenesis. A germline stem cell produces a cystoblast which undergoes four rounds of pre-meiotic mitosis to form a cyst consisting of 16 cells. In region 2a, up to four cells in a cyst initiate meiosis and form the SC. In region 2b, two cells (pro-oocytes) maintain the meiotic state. By region 3, one of these cells is finally selected as the oocyte, and all other cells have become nurse cells. Karyosome forms at stage 2–3 of oogenesis. At stage 3 and later, the SC gradually disassembles from chromosome arms, except in centromeric regions. At stage 14, the oocyte completes maturation and arrests in meiotic metaphase I. (B) The spatial distribution and quantified level of H3K4me3 in control and *Kdm5/lid* RNAi oocytes. The total H3K4me3 signal intensity was significantly higher in *Kdm5/lid* RNAi oocytes at all stages (p<0.001). n≥7. Error bars represent the standard errors of the mean. reg; region, st; stage. (C) Karyosome defects caused by two different shRNAs (*lid* RNA1 and *lid* RNAi2) and a *Kdm5/lid* mutant (*lid*^*10424/k06801*^). The karyosome morphologies at stages 3–9 were classified into three categories: "spherical" when the karyosome shows a spherical shape, "mildly distorted" when spherical shape was distorted but largely maintained, and "distorted" when spherical shape was largely disrupted. *** indicates a significant difference from the control (p<0.001). n≥18. (D) γH2Av foci which mark DSBs in meiotic nuclei in region 2a, 2b and region 3 of control and *Kdm5/lid* RNAi ovaries. DSBs are repaired by region 3 in both. Meiotic nuclei were identified by C(3)G staining. n≥12. (E) Karyosome morphology at stage 4/5 in control RNAi, *Rad51/spnA*, *Kdm5/lid RNAi*, *Chk2/mnk*^*p*6/+^ *spnA* double mutant, *Chk2/mnk*^p6/+^ mutant with *Kdm5/lid* RNAi, and *Kdm5/lid p53* double RNAi. The frequency of abnormal karyosomes at stage 3–9 was quantified for each genotype. n≥16. Suppression of meiotic checkpoint does not rescue the karyosome defect of *Kdm5/lid* RNAi (p = 1.00). Scale bars = 5 μm.

In wild-type *Drosophila* prophase I oocytes, meiotic chromosomes cluster together to form a spherical structure, the karyosome, after completion of recombination ([18]; **Fig 1A and 1C**). Through an RNAi screen of nuclear and chromatin-binding proteins [20], we identified *Kdm5/little imaginal discs* (*lid*) encoding a histone demethylase as a gene important for normal karyosome morphology. Expression of short hairpin RNA (shRNA) targeting *Kdm5/lid* in female germlines throughout oogenesis resulted in sterility and abnormal morphology of the karyosome in most oocytes (**Fig 1C**). To exclude the possibility of off-target effects, another shRNA targeting *Kdm5/lid* was used and showed a similar karyosome defect (**Fig 1C**). Furthermore, a semi-lethal *Kdm5/lid* mutant (*lid*^*10424/k06801*^; [21]) showed a similar karyosome defect in oocytes, confirming that Kdm5/Lid is required for proper karyosome morphology (**Fig 1C**).

*Kdm5/lid* is known to genetically interact with another histone demethylase gene, *lsd1*, by antagonising each other in position effect variegation [22]. To test for a genetic interaction in meiosis, we co-depleted this protein together with Kdm5/Lid. We did not see enhancement or rescue of the *Kdm5/lid* RNAi prophase I defect by co-RNAi of *lsd1* (**S1 Fig**).

### Kdm5/Lid is a major H3K4me3 demethylase in oocytes

Kdm5/Lid and Kdm2 are two histone demethylases thought to remove a methyl group from H3K4me3 in *Drosophila* [23]. To determine the contribution of Kdm5/Lid activity in oocytes, we probed the level and distribution of H3K4me3 in the oocyte nucleus at each oogenesis stage by immunostaining using a specific antibody against this modification.

The area of ovarioles in which an oocyte is determined (called the germarium) is subdivided into regions 1, 2a, 2b and 3, according to the morphology and developmental events (**Fig 1A**; [24]). In region 1, a cystoblast produced by a germline stem cell undergoes four rounds of pre-meiotic mitosis to generate a cyst containing 16 cells. Up to four cells in each cyst initiate meiosis in region 2a and, among them, two (pro-oocytes) maintain the meiotic state in region 2b, and one cell is finally selected as the oocyte in region 3. Subsequently, oogenesis is divided into stages 1–14 (stage 1 corresponds to region 3). In control meiotic nuclei, H3K4me3 signals were observed on chromosomes except for a DAPI-intense region which corresponds to pericentromeric heterochromatin ([25]; **Fig 1B**). The total signal intensity was gradually decreased as oogenesis progressed. In *Kdm5/lid* RNAi, intense H3K4me3 signals were observed on chromosome arms excluding pericentromeric heterochromatin, and the level of H3K4me3 was much higher than seen in the control RNAi throughout oogenesis (**Fig 1B**). Therefore, Kdm5/Lid provides a major H3K4 demethylase activity during meiosis.

### Depletion of Kdm5/Lid affects karyosome morphology independently from the meiotic recombination checkpoint

H3K4me3 is associated with DNA double strand breaks (DSBs) induced by Spo11 to initiate the recombination process in mice and yeast [10,11]. Furthermore, it is known that persistent DSBs lead to a karyosome defect through activation of meiotic recombination checkpoint signalling in *Drosophila* oocytes [26]. Therefore, increased H3K4me3 level may result in persistent DSBs which activate the meiotic checkpoint signalling and in turn induces karyosome defects.

To test this possibility, DSBs were detected by immunostaining using an antibody [27] against phosphorylated H2Av (the H2AX homologue) that is associated with DSBs (**Fig 1D**). In both control and Kdm5/Lid-depleted meiotic nuclei, DSBs were observed in region 2a and 2b, and nearly disappeared by region 3 as previously reported ([28]; **Fig 1D**). Therefore, it indicates that the karyosome defect observed in Kdm5/Lid-depleted oocytes is not due to a checkpoint-triggering delay in the repair of DSBs. Furthermore, the main karyosome defect in *Kdm5/lid* RNAi or mutant is disruption of its spherical morphology. In contrast, DSB-repair mutants typically show chromatin association with the nuclear envelope [27].

To test whether the karyosome defect in *Kdm5/lid* RNAi is mediated by the meiotic recombination checkpoint, the checkpoint signalling was suppressed by a mutation in the checkpoint kinase Chk2/Mnk. The karyosome defect caused by a mutation in a DNA repair gene (*Rad51/spnA*) was rescued by the *mnk* mutation as previously shown ([29]; **Fig 1E**). In contrast, the karyosome defect in *Kdm5/lid* RNAi was not rescued by the *mnk* mutation (**Fig 1E**). Furthermore, co-depletion of an Mnk effector protein, p53 [26,30], did not rescue the karyosome defect in *Kdm5/lid* RNAi (**Fig 1E**). These results demonstrated that the karyosome defect caused by *Kdm5/lid* RNAi is independent of the meiotic recombination checkpoint, in accordance with the timely DSB repair observed (**Fig 1D**).

### Maintenance of centromere clustering is disrupted upon loss of Kdm5/Lid

**Fig 2:**
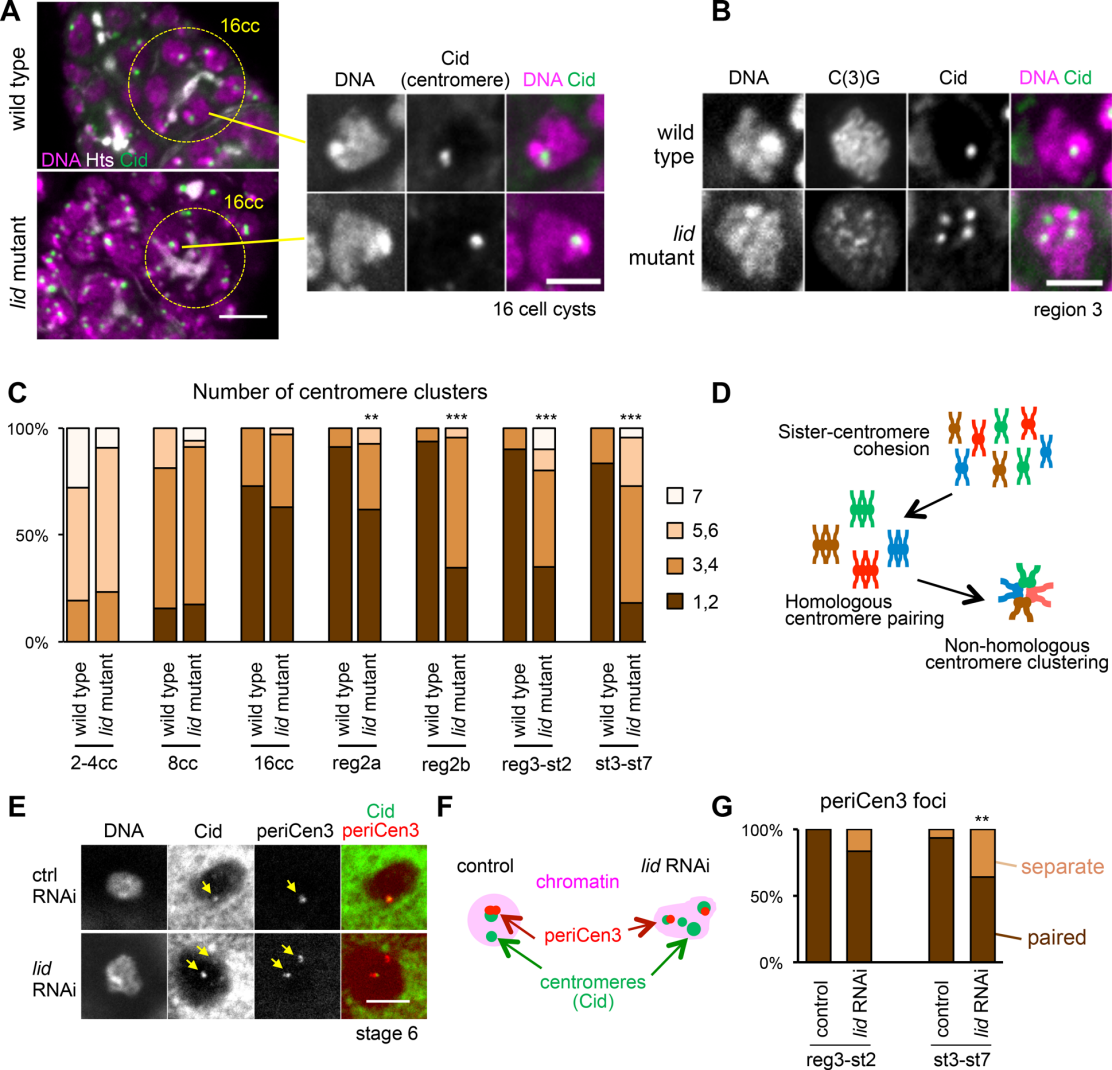
Homologous centromere pairing is disrupted upon loss of Kdm5/Lid. (A) Centromere clustering in pre-meiotic nuclei of 16-cell cysts (16 cc) in wild-type and *Kdm5/lid* mutant ovaries. Parts of the germarium containing 16-cell cyst (circled) are shown in the left panels (Scale bar = 5 μm), and magnified images of one cell, in each case, are shown in the right panels (Scale bar = 3 μm). The anterior end of the germarium is oriented towards the top. The stage of each cyst was determined using the morphology of fusome visualised by Hts. (B) Increased number of centromere clusters in *Kdm5*/*lid* mutant oocytes in comparison to wild type in region 3. The SC component C(3)G was used to identify oocytes. Scale bars = 3 μm. (C) The number of centromere clusters in pre-meiotic and meiotic nuclei in various oogenesis stages of wild type and the *Kdm5/lid* mutant. cc; cell cyst, reg; region, st; stage. ** and *** (p<0.01 and 0.001) indicate significant differences from wild type in terms of the frequency of nuclei with one or two centromere clusters. ≥32 nuclei and meiotic nuclei were quantified for each pre-meiotic stage and region 2a, while ≥16 germaria were quantified for each later meiotic stage. (D) A schematic diagram showing three levels of centromere association in oocytes, cohesion of sister-centromeres, pairing of homologous centromeres and clustering of non-homologous centromeres. (E) Closely paired signals of the pericentromeric dodeca satellite specific to chromosome 3 (periCen3) that co-localise with CenpA/Cid foci in the wild-type oocyte at stage 6, and which are clearly separated into two foci in the *Kdm5/lid* RNAi oocyte at stage 6. Arrows indicate Cid and periCen3 foci. Scale bars = 5 μm. (F) Schematic representation of the behaviour of the pericentromere 3 signals (periCen3) in control and *Kdm5/lid* RNAi oocytes. (G) The proportion of paired or separate pericentromeric signals (periCen3) in control and *Kdm5/lid* RNAi oocytes. Two signals separated by ≥1 μm were defined as "separate" in this quantification. ** indicates significant difference from the control (p<0.01). n≥17.

To determine the chromosome organisation within the karyosome, the location of centromeres was assessed by immunostaining of the centromeric protein CenpA/Cid (**Fig 2A–2C)**. In control germaria, clustering of centromeres started around the pre-meiotic 8-cell stage (**Fig 2A–2C)**. By region 2a, centromeres were clustered to one or two closely located foci in nearly all meiotic nuclei (**Fig 2A–2C)**, which is consistent with previous observations [14–17]. In *Kdm5/lid* mutant or RNAi germaria, centromeres were clustered in region 1 with the normal timing, but showed a clustering defect in region 2a or later (**Fig 2A–2C; S2A–S2C Fig**). This indicates that Kmd5/Lid is required for maintenance of centromere clustering during meiosis, but not for establishment during pre-meiotic stages.

There are three levels of centromere association observed in control oocytes, which are cohesion of sister-centromeres, pairing of homologous centromeres, and clustering of non-homologous centromeres (**Fig 2D**; [13]). To determine which level of association is disrupted in *Kdm5/lid* RNAi, fluorescence *in situ* hybridisation was carried out using the pericentromeric dodeca satellite, specific to chromosome 3, as a probe. Control oocytes have only one focus in the nucleus, indicating that sister-centromeres were joined by cohesion and homologous centromeres were paired properly (**Fig 2E–2G**). About half of the *Kdm5/lid* RNAi oocytes showed two separate foci, the remainder having one focus. Importantly no oocytes had more than two foci (**Fig 2E–2G**).

We further examined pairing of pericentromeric regions of chromosome 2 and X.

The pericentromeric AACAC satellite specific to chromosome 2 showed a pairing defect similar to that seen using the chromosome 3 pericentromeric probe (**S2D Fig**). In contrast, pericentromeric 359-bp repeats specific to the X chromosome did not show a pairing defect (**S2E Fig**). This is in agreement with previous reports showing that X-chromosome pairing is mediated by a different mechanism from chromosome 2 and 3 [16,17]. Single RNAi of *lsd1* encoding another demethylase did not display a centromere clustering defect or pairing defect of the pericentromeric satellites of chromosome X or 3 (**S1A, S1C and S1D Fig**).

These results showed that cohesion of sister-centromeres was maintained, but pairing of homologous pericentromeric regions of chromosomes 2 and 3 was disrupted by Kdm5/Lid depletion.

### Kdm5/Lid is required for maintaining the SC along chromosome arms but not for localisation of the transverse protein C(3)G at centromeres

**Fig 3:**
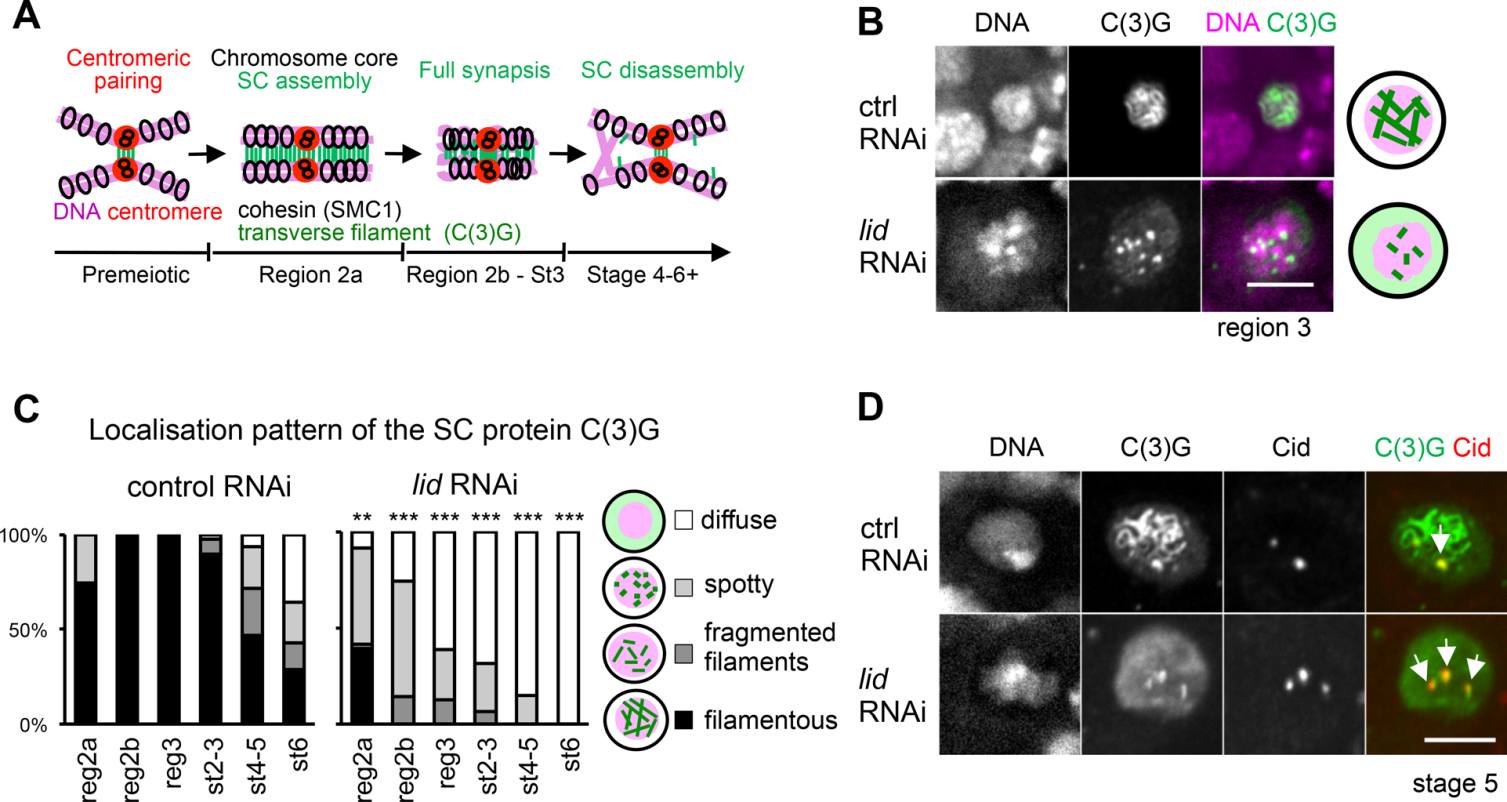
Loss of Kdm5/Lid leads to partial formation and instability of the SC along chromosome arms but does not affect persistence of a transverse protein at centromeres. (A) Schematic representation of early meiotic events. In region 2a, the SC starts to assemble to promote synapsis which results in crossover and chiasmata formation. The filamentous structure of the SC mostly disassembles by stage 6 of oogenesis except at centromeres. (B) A control region-3 oocyte with filamentous C(3)G staining, and a *Kdm5/lid* RNAi oocyte with spots of C(3)G staining. (C) Quantification of C(3)G staining pattern in control and *Kdm5/lid* RNAi oocytes. For region 2a, we classified each germarium based on the majority of the SC morphology in multiple nuclei which accumulate C(3)G. For region 2b where the two nuclei accumulate C(3)G, each germarium was scored for the morphology of the better formed SC. ** and *** indicate significant differences in the pattern distribution from control (p<0.01 and p<0.001, respectively). n≥14. (D) Centromeric C(3)G localisation in control and *Kdm5/lid* RNAi oocytes at stage 5. Cid, the *Drosophila* CenpA, highlights all centromeres. The arrows indicate colocalisation of the Cid signals with centromeric C(3)G. Colocalisation was observed in all oocytes examined (n = 27 for stage 4–6). Scale bars = 5 μm.

The SC is an elaborate proteinaceous structure formed in early prophase I (prior to karyosome formation; **Figs 1A and 3A**) that stabilises pairing of homologous chromosomes and promotes crossover between them. Loss of the transverse protein C(3)G, which bridges two homologues in the SC is known to result in a loss of homologous centromere pairing [31,14]. In the premeiotic 8-cell stage in the germarium, SC components accumulate at centromeres [14,32,17]. Once the 16-cell stage enters meiotic zygotene in region 2a, the SC is visible in punctae in up to four nuclei, which form a full-length SC upon entering pachytene in region 2a. In region 2b, fully assembled filamentous SC is restricted to the two pro-oocytes and subsequently, only the oocyte retains a full SC in region 3 [33]. The SC persists on the karyosome in the oocyte until stage 6, when the filaments appear mostly broken and in short threads [33,34]. To determine whether Kdm5/Lid is important for the integrity of the SC, we immunostained the transverse protein C(3)G in ovaries expressing the control or *Kdm5/lid* shRNA (**Fig 3; S3 Fig**).

In control, filamentous C(3)G accumulated on chromosome arms in multiple nuclei at the earliest meiotic region 2a (**S3A Fig**). By region 2b, the filamentous C(3)G structure was formed fully along chromosome arms in one of two pro-oocytes (**Fig 3A–3C**). This fully formed filamentous structure in the oocyte started fragmenting at stage 3, and had fragmented or disassembled in the majority of oocytes by stage 6, except in the centromeric regions where C(3)G remained associated (**Fig 3A, 3C and 3D; S3B Fig**).

In region 2a, filamentous C(3)G structures were observed in less than half of the *Kdm5/lid* RNAi nuclei accumulating C(3)G, but by region 2b, these filamentous structures were fragmented (**Fig 3B and 3C**). By stage 4, all C(3)G structures were disassembled and dissociated from chromosome arms, much earlier than in control oocytes (**Fig 3C and 3D**). However, C(3)G remained associated with centromeric regions, as seen in control oocytes (**Fig 3D**).

These observations point to a role for Kdm5/Lid in stabilising or maintaining the SC along chromosome arms, although it is dispensable for association of C(3)G with centromeric regions.

### Kdm5/Lid is required for proper formation and maintenance of chromosome cores, not persistence of the centromeric cohesin

**Fig 4:**
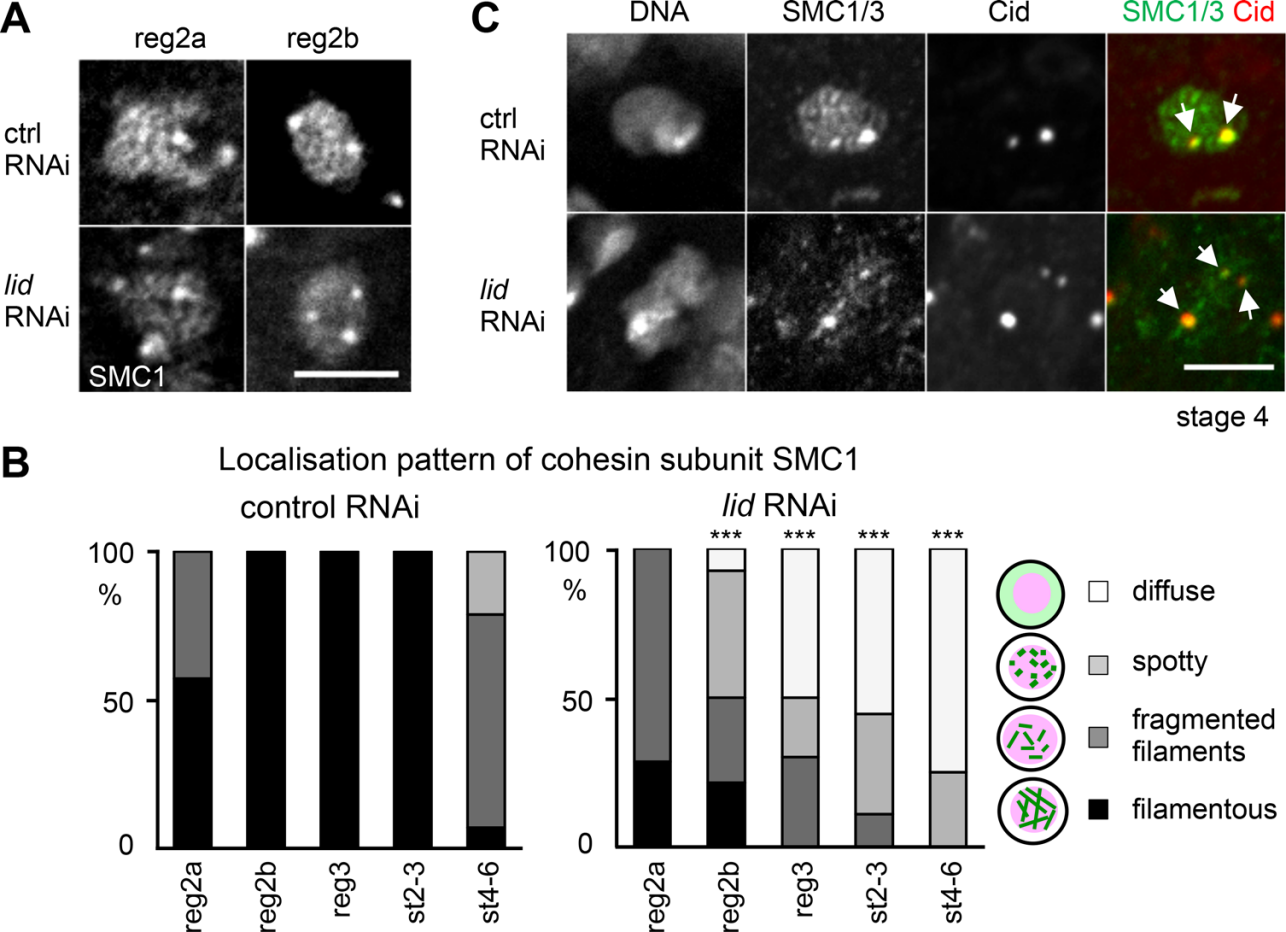
Loss of Kdm5/Lid leads to instability of chromosome cores along arms but does not affect persistence at centromeres. (A) SMC1 signals in meiotic nuclei in region 2a and 2b of control and *Kdm5/lid* RNAi ovaries, showing filamentous patterns in control, and fragmented filaments in region 2a and mainly diffuse pattern in region 2b of *Kdm5/lid* RNAi ovaries. (B) Quantification of SMC1 staining pattern in control and *Kdm5/lid* RNAi meiotic nuclei. *** indicates a significant difference in the pattern distribution from the control (p<0.001). n≥7. Chromosome cores visualised by filamentous SMC staining fails to be maintained in *Kdm5/lid* RNAi. (C) Centromeric SMC1/3 localisation in control and *Kdm5/lid* oocytes at stage 4. Colocalisation between Cid and SMC1/3 foci was observed in all oocytes examined (n = 13 for stage 4–6). Arrows indicate Cid and SMC1/3 foci. Scale bars = 5 μm.

Defective C(3)G filaments in meiotic nuclei depleted of Kdm5/Lid may be caused by defects in the underlying chromosome cores, filamentous cohesin-containing structures visible under a microscope due to shortening and compaction of chromosome arms (**Fig 3A**; [6]). To determine whether the chromosome core is disrupted by *Kdm5/lid* RNAi, the cohesin subunit SMC1/3 was visualised by immunostaining (**Fig 4; S4 Fig**).

In control meiotic nuclei, the filamentous structure of cohesin was mostly assembled by region 2a, and fully by region 2b (**Fig 4A and 4B**). These filamentous structures started disassembling at stage 4 (**Fig 4B and 4C**). In meiotic nuclei depleted of Kdm5/Lid, the filamentous structure of cohesin was assembled partially by region 2a in a similar way to control, but started being disassembled at region 2b and was mostly disassembled by stage 4 (**Fig 4**). However, we noticed that the cohesin signal remained associated with centromere regions (**Fig 4C**), much like our observation of persisting C(3)G staining at centromeres (**Fig 3D**).

These results suggest chromosome cores are partially assembled, but are either unstable or precociously disassembled from chromosome arms in the absence of Kdm5/Lid.

### The demethylase activity of Kdm5/Lid is not required for prophase I chromatin organisation

Li et al. (2010) previously proposed that the demethylase activity of Kdm5/Lid is not required for viability and fertility [23], suggesting a demethylase-independent function of Kdm5/Lid. To test whether the demethylase activity is essential for the nuclear organisation of prophase I, the wild-type gene and the *Kdm5/lid* gene with the mutated catalytic site (JmjC*; H637A, E639A; [35]) under its own promoter were obtained from the authors. Transgenic flies were generated and tested for the ability to rescue the phenotype of the *lid*^*10424/k06801*^ mutation.

**Fig 5:**
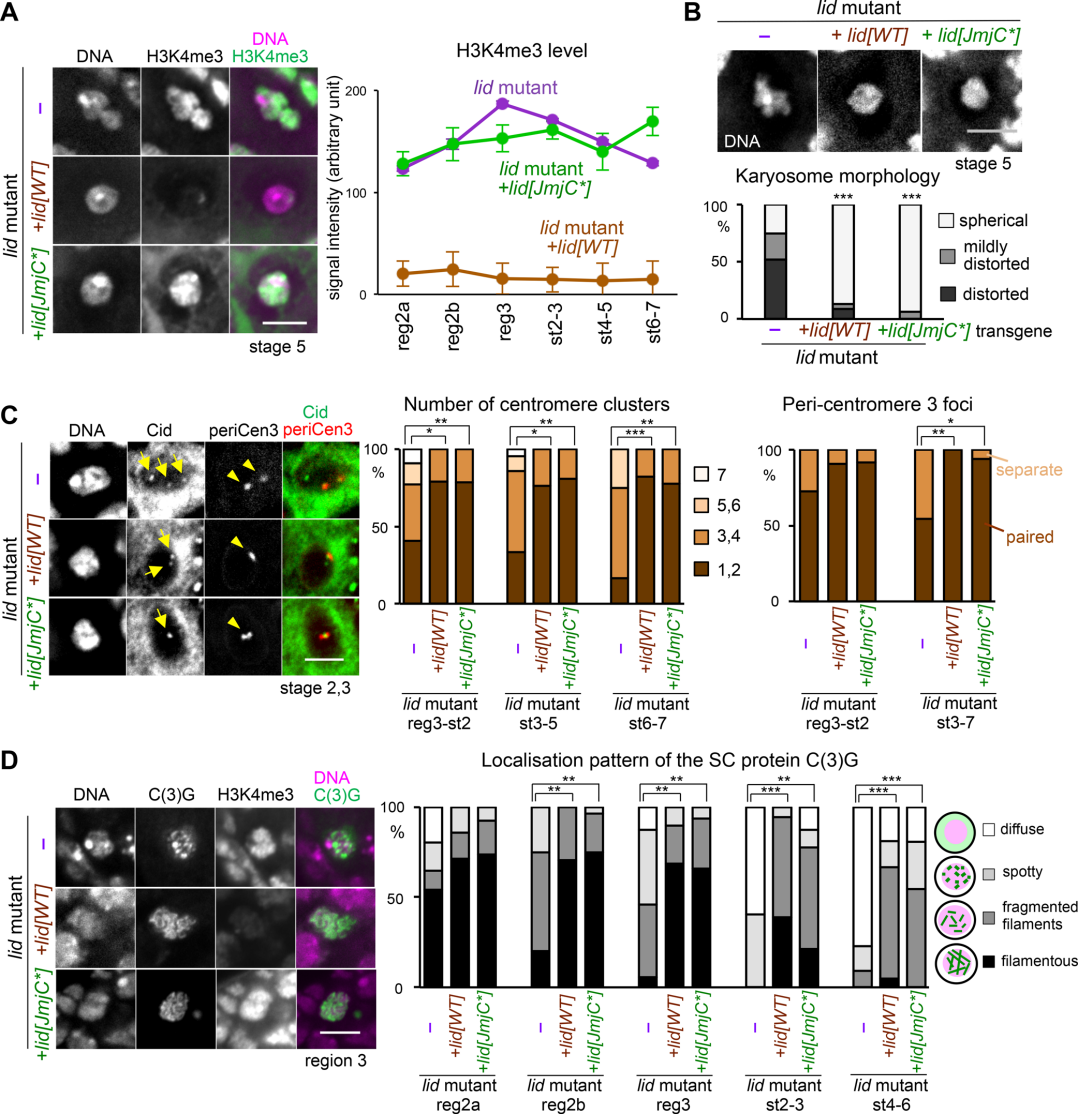
Kdm5/Lid demethylase activity is dispensable for meiotic chromatin reorganisation. (A) Distribution and intensity of H3K4me3 on meiotic chromosomes in a *Kdm5/lid* mutant (*lid*^*10424/k06801*^) without a transgene (–), the *Kdm5/lid* mutant carrying a wild-type transgene (*lid[WT])* and *the Kdm5/lid* mutant carrying a catalytically inactive transgene (lid[JmjC*]). Images of the karyosome in stage-5 oocytes were taken and the contrast has been enhanced using identical conditions. The total signal intensity of H3K4me3 on the karyosome was measured as described in Materials and Methods. Error bars indicate standard errors. n≥6. The signal intensity in the *Kdm5/lid* mutant carrying no transgenes (–) or the lid[JmjC*] transgene is significantly different from the one carrying the *lid[WT]* transgene at all stages (p<0.001). (B) Rescue of karyosome defects of the *Kdm5/lid* mutant by both the wild-type transgene (*lid[WT]*) and demethylase-inactive transgene (lid[JmjC*]). Karyosome morphology was observed in the *Kdm5/lid* mutant oocytes carrying no transgenes (–), the *lid[WT]* transgene or the lid[JmjC*] transgene, and was classified as in Fig 1C. *** indicates a significant difference in the pattern of distribution from the control (p<0.001). n≥18. (C) Centromere pairing and clustering do not require the demethylase activity of Kdm5/Lid. Centromeres highlighted by the Cid antibody are indicated by arrows, and foci of pericentromeric dodeca satellite specific to chromosome 3 (periCen3) are indicated by arrowheads. The numbers of centromere (Cid) clusters were counted for each group of stages (n≥12). Oocyte nuclei with tightly paired (<1 μm) or separate (≥1 μm) periCen3 foci were counted for each group of stages (n≥11). ***, ** and * indicate significant differences (p<0.001, p<0.01 and p<0.05, respectively). (D) SC morphology is not affected by loss of the Kdm5/Lid demethylase activity. Filamentous structures of C(3)G observed in region 3 oocytes from the *Kdm5/lid* mutant carrying the *lid[WT]* or lid[JmjC*] transgene, and a spotty appearance observed in the *Kdm5/lid* mutant alone. The SC component C(3)G and H3K4me3 were co-stained, imaged and contrast-enhanced using identical conditions. The morphology of the C(3)G-containing structure was categorised and counted for each group of stages (n≥10). ***, ** and * indicate significant differences (p<0.001, p<0.01 and p<0.05, respectively). Scale bars = 5 μm in all images.

As expected, the *Kdm5/lid* mutant carrying a catalytically inactive *Kdm5/lid* transgene (JmjC*) showed high levels of H3K4me3 in oocytes, similar to the *Kdm5/lid* mutant without a transgene, while the *Kdm5/lid* mutant carrying a wild-type *Kdm5/lid* transgene had low levels of H3K4me3 (**Fig 5A**). This confirmed that the mutation indeed abolishes the demethylase activity.

The *Kdm5/lid* mutant without a transgene showed a similar phenotype as *Kdm5/lid* RNAi in terms of karyosome defects (**Fig 5B**), unclustering of centromeres (**Fig 5C**) and a failure to maintain the SC along the arms (**Fig 5D**). Introduction of a wild-type *Kdm5/lid* transgene rescued all of these defects in the *Kdm5/lid* mutant (**Fig 5B–5D**). Remarkably, the *Kdm5/lid* mutant carrying the catalytically inactive transgene (JmjC*) showed largely normal karyosome morphology (**Fig 5B**), centromere clustering (**Fig 5C**) and SC morphology (**Fig 5D**). High H3K4me3 levels and normal SC morphology were observed in the same germarium, demonstrating that catalytically inactive Kdm5/Lid rescued the synaptonemal complex defects of the *Kdm5/lid* mutant without changing the H3K4me3 levels (**Fig 5D**).

Taken together, we conclude that the demethlyase activity of Kdm5/Lid is dispensable for chromatin organisation in meiotic prophase I.

### Depletion of Kdm5/Lid affects chromosome alignment in prometa/metaphase I, as a potential consequence of reduced crossing-over

**Fig 6:**
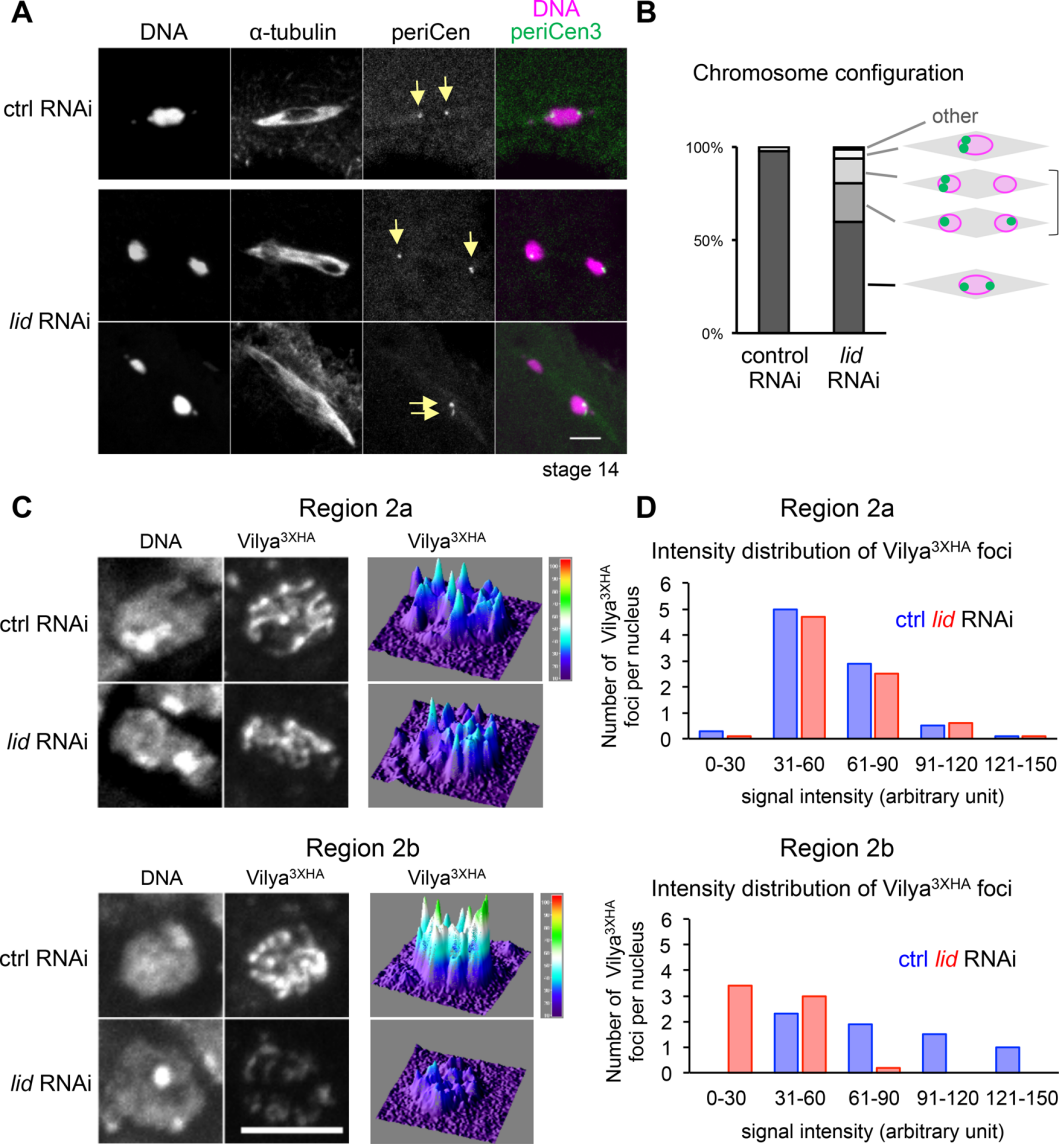
Abnormal chromosome positioning and orientation in prometa/metaphase I and a potential reduction in crossovers in oocytes lacking Kdm5/Lid. (A) Mis-positioned chromosomes with normal spindle morphology in *Kdm5/lid* RNAi oocytes in prometa/metaphase I in comparison to control RNAi. Chromosome orientation was assessed by *in situ* hybridisation using dodeca satellite as a pericentromere 3 probe (arrows). Scale bar = 5 μm. (B) Quantification of chromosome configuration in control and *Kdm5/lid* RNAi oocytes. The “other” category includes a meiotic figure with more than two foci of the pericentromere 3 signal. The frequency of mis-oriented pericentromere 3 in *Kdm5/lid* RNAi is significantly different from the control RNAi (p<0.05). n≥45. (C) Localisation and signal intensity of Vilya-3xHA foci in meiotic nuclei in region 2a and 2b of control and *Kdm5/lid* ovaries expressing HA-tagged Vilya. Scale bar = 5 μm. (D) The signal intensity of Vilya^3xHA^ foci in region 2a and 2b meiotic nuclei of control and *Kdm5/lid* RNAi ovaries expressing HA-tagged Vilya. The graphs show the numbers of foci per meiotic nucleus with the maximum signal intensity in indicated ranges. Foci with a signal intensity lower than 30 are significantly more frequent in *Kdm5/lid* RNAi than in control (p<0.001). Intensities of ≥66 Vilya^3XHA^ foci have been measured for each region of each genotype.

To see the effects of Kdm5/Lid depletion in chromosome segregation at later stages, mature oocytes that mainly arrest in metaphase I were subjected to fluorescence *in situ* hybridisation using the pericentromeric dodeca satellite probe specific to chromosome 3, and further stained for DNA and α-tubulin (**Fig 6A and 6B**). Control oocytes had a cluster of meiotic chromosomes associated with a bipolar spindle. Two pericentromeric foci were observed at the edge of the chromosome cluster (**Fig 6A and 6B**). This represents the configuration in which sister centromeres were tightly associated together, but homologous centromeres were pulled towards the opposite poles. Homologous chromosomes are linked through chiasmata which are the result of crossovers in prophase I.

A significant proportion of *Kdm5/lid* RNAi oocytes had two separate clusters of meiotic chromosomes (sometimes of unequal sizes) located closer to the poles of the bipolar spindle. In some cases, both clusters contained one centromere 3 signal each, while in the other cases, one of the chromosome clusters contained both centromere 3 signals (**Fig 6A and 6B**). Therefore, Kdm5/Lid is crucial for chromosome positioning and orientation in metaphase I oocytes.

Such chromosome positioning near the spindle poles resembles achiasmatic chromosomes in mutants which do not undergo meiotic recombination [36]. To gain further insights into whether Kdm5/Lid depletion affects crossing-over, we examined the localisation of the newly discovered Vilya protein [37]. In wild type, Vilya has been shown to associate weakly with the SC, and to further accumulate strongly at most DSBs in region 2a and at foci which are suggested to be crossover sites in region 2b.

It was challenging to confidently distinguish Vilya accumulation at crossover sites from Vilya localisation to fragmented SC commonly seen in *Kdm5/lid* RNAi pro-oocytes in region 2b. Therefore we counted the number of Vilya foci in region 2a and 2b meiotic nuclei and measured the signal intensity of each focus (**Fig 6C and 6D**). In region 2a, the number and intensity of the foci was nearly identical between *Kdm5/lid* RNAi and control (**Fig 6C and 6D**), indicating Kdm5/Lid does not affect DSB formation consistent with our previous observations of phospho-H2Av. In region 2b, the intensity of most foci was much weaker in *Kdm5/lid* RNAi pro-oocytes than control (**Fig 6C and 6D**). These weak foci may represent crossover sites that accumulate Vilya at a low level, or reflect reduction of Vilya signals due to underlying SC defects. However, we favour the alternative interpretation in which most of these weak foci represent Vilya localisation to fragmented SC, and that fewer crossovers are present in *Kdm5/lid* RNAi pro-oocytes. This is in good agreement with our observation that *Kdm5/lid* RNAi oocytes show abnormal metaphase I chromosome positioning similar to achiasmatic chromosomes, and with a proposed role of the SC in promoting crossing-over between homologues in various organisms [5].

## Discussion

In this article we show that the histone demethylase Kdm5/Lid controls maintenance of both the chromosome axis and the synaptonemal complex (SC). Loss of Kdm5/Lid leads to an increase of H3K4me3 levels on chromosome arms, demonstrating major demethylase activity for Kdm5/Lid in the female germline. Euchromatic, but not centromeric cohesin and SC proteins are lost in the absence of Kdm5/Lid, with a pronounced unclustering of centromeres and disruption of karyosome morphology throughout oogenesis. Meiotic DNA double strand breaks were formed and repaired normally. Unexpectedly, the demethylase activity of Kdm5/Lid is not required for maintaining the SC, centromere clustering or the proper formation of the karyosome. Our data lead us to propose Kdm5/Lid to act as a regulator of early meiotic chromatin reorganisation independently of its demethylase activity. In the absence of Kdm5/Lid, fewer strong foci of crossover-associated Vilya are found and consequently alignment of bivalents in meiosis I is compromised. Consistent with its role in maintenance of the SC, our data suggest that Kdm5/Lid promotes crossing over and therefore ensures accurate chromosome segregation.

Our crucial finding is the requirement of Kdm5/Lid for chromatin organisation of meiotic prophase I nuclei. Chromatin architecture dramatically changes during meiosis. The roles of structural components, such as cohesins and SC components, have been well studied. In contrast, how the chromatin architecture is regulated is not well understood, except for an involvement of master-regulator kinases such as Plk, NHK-1 or the chromosomal passenger complex [19,38–41]. Histone modifying enzymes are good candidates to connect chromatin structure to chromosomal regulator complexes. For example, there is clear evidence for H3K4me3-enrichment at meiotic recombination sites in several organisms, and in human and mice this is promoted by DNA-binding of PRDM9, to specify “hotspots” of DSB-mediated recombination initiation [9–12,42].

The mutant phenotype of *Kdm5/lid*, which is phenocopied by RNAi, is distinct from other known meiotic mutants affecting prophase I complexes in *Drosophila*. We found that the SC and its chromosome core only partially formed and failed to be maintained along chromosome arms, compromising synapsis. Interestingly, judged from the accumulation of the transverse SC element C(3)G and the cohesin core subunits SMC1/3, both complexes are established and maintained at centromeric regions. In mutants for the *Drosophila*-specific cohesin proteins ORD, SOLO and SUNN, despite similarities in disassembling the core and SC, centromeric SMC is not maintained [6,43–45]. Thus, our study, together with the previous data, support the idea that cohesin at centromeres and chromosome arms is controlled differently [6].

Strikingly, premeiotic clustering of centromeres is established in the absence of Kdm5/Lid, and only when entering meiosis, the nuclei displayed unclustering of centromeres, which is distinct from SC mutants which show unclustering of centromeres [16,17]. This raises the possibility that centromere clustering in premeiotic mitosis and meiosis may be regulated independently.

Another interesting observation is that despite persistent accumulation of C(3)G and SMC1/3 at centromeres, homologous centromeres of chromosome 2 and 3 have become unpaired without Kdm5/Lid. As expected, the X chromosome, which appears to employ a separate mechanism of pairing [16,17], is not affected. Sister-centromeres are still held together, which is a similar phenotype to *c(3)G* mutants but distinct from cohesion defects seen in other mutants [14,15,44–47]. It was thought that the persistent pairing of homologous centromeres until spindle formation is important for accurate chromosome segregation, and that this is mediated by centromere localisation of SC components. Our results suggest either that Kdm5/Lid function is important for making these centromeric components functional, or that maintaining the SC at chromosome arms is important for maintaining homologous centromere pairing.

Furthermore, Kdm5/Lid is important for karyosome formation. The karyosome is a compact spherical body made of clustered chromosomes formed within the oocyte nucleus after recombination has completed [18]. A similar structure is also found in oocytes of other animals, including humans [48]. The karyosome defect is likely to be separable and independent from the SC defect, as mutants defective for SC formation do not show karyosome defects [14], and the SC is disrupted before the karyosome is formed. This suggests that Kdm5/Lid is a more general regulator of the chromatin architecture in oocytes. It is known that a failure of DSB repair would result in abnormal karyosome morphology and a delay in SC disassembly through activating meiotic recombination checkpoint signalling [25, 27]. However, the two following observations argue against the possibility that a delay in DSB repair causes the karyosome defects seen in *Kdm5/lid* RNAi. Firstly, formation and repair of DSBs take place in a timely fashion without Kdm5/Lid. Secondly, suppression of the meiotic recombination checkpoint did not rescue the karyosome defects in *Kdm5/lid* RNAi.

What would be the consequence of these defects? In a proportion of metaphase I oocytes lacking Kdm5/Lid, chromosomes are abnormally located closer to the spindle poles with or without separation of homologous chromosomes. Location of two chromosome masses, with occasionally unequal sizes, close to the poles is similar to observations in *ord* mutants, which fail to maintain chiasmata and to arrest at metaphase I [46]. Additionally, an error in bi-orientation may be caused by such an unpairing of homologous centromeres, as homologous centromeric pairing is proposed to promote disjunction of homologues at anaphase I (reviewed in [13]). Indeed, pachytene nuclei lacking Kdm5/Lid have fewer strong foci of Vilya which has been suggested to mark crossover sites [37]. Although the interpretation is tricky, it is tempting to speculate that a failure in maintaining the SC may lead to a reduced number of crossovers. This in turn would result in metaphase chromosomes lacking chiasmata, and thus failing to maintain a physical link on the metaphase plate.

We showed that Kdm5/Lid provides a major demethylase activity towards H3K4me3 in prophase I oocytes, as its loss dramatically increased levels of H3K4me3, in accordance with previous observations of global increases after Kdm5/Lid depletion [49]. A potential contribution of the second demethylase, Kdm2, remains to be determined. The H3K4me3 epigenetic marker is often associated with transcriptionally active genes [50]. However, we found that catalytically inactive Kdm5/Lid can perform its function in SC maintenance, centromere clustering/pairing and karyosome formation in oocytes. It was previously shown that Kdm5/Lid has transcriptional roles independent of its catalytic activity in mammals and *Drosophila* [35,23,51], but specific functions had not been identified at the cellular level. Importantly, the rescue of Kdm5/Lid loss by catalytically inactive Kdm5/Lid is not mediated through a removal of the methyl group from H3K4me3, for example by activation of the other demethylase Kdm2 or by residual Kdm5/Lid, as H3K4me3 was abnormally high in the presence of the demethylase-dead transgene, comparable to levels seen upon loss of Kdm5/Lid.

If Kdm5/Lid's catalytic activity is not essential, how does Kdm5/Lid regulate chromatin architecture? Previous studies have already hinted at such mechanisms. Kdm5/Lid has been shown to form a complex with other proteins including the histone deacetylase Rpd3, and to negatively regulate activity of Rpd3 [52]. An increase in histone acetylation is observed in mouse oocytes reaching mature stages [53], and the inhibition of Rpd3’s deacetylation of target genes by Kdm5/Lid could possibly contribute to chromatin accessibility and structure in meiosis. In line with this, in cultured cells, Kdm5/Lid overexpression increases acetylation levels of H3K9, an Rpd3 target site [54,55], whereas Kdm5/Lid-loss reduces H3K9Ac [55]. Thus, we assessed if co-depleting Rpd3 from ovaries restores the observed defects upon Kdm5/Lid depletion. Unfortunately, double RNAi of Rpd3 and Kdm5/Lid resulted in tiny ovaries which we were not able to analyse. It is further possible that Kdm5/Lid may be required for the activity or localisation of other subunits in this complex, which is essential for chromatin organisation in prophase I oocytes.

In mammals the Kdm5 family is linked with multiple processes, including cellular senescence, cell differentiation and mitochondrial biogenesis [56–61]. The Kdm5 functions are often assumed to be mediated by transcriptional regulation, as H3K4me3 is thought to be a transcription-active epigenetic marker. However, in most cases, requirement of the catalytic activity has not been demonstrated or tested. Our finding emphasises the chromatin regulating functions of Kdm5 independent from its catalytic activity, over the conventional view of Kdm5 as an "eraser" of the epigenetic mark.

Finally, the Kdm5 family of proteins function in diseases including tumorigenesis and mental retardation [62–71]. Our findings may provide a new insight into how malfunctions in this family of histone demethylases causes disease at the molecular and cellular levels.

## Materials and Methods

### *Drosophila* genetics

Standard fly techniques were followed [72]. All fly stocks have been cultured at 25°C in standard cornmeal media. For RNAi in ovaries, *P{Gal4*::*VP16-nos*.*UTR}MVD1* were crossed with the following RNAi TRiP lines (Harvard Medical School): *ctrl RNAi* (*white*; GL00094), *lid RNAi* (*GLV21071*, *GL00612*), *lsd1 RNAi (HMS00638)*, *p53 RNAi (GL01220)*, *lid* mutant lines with a P-element insertion in the 5’UTR *(y*^*1*^ *w*^*67c23*^*; P{lacW}lid*^*k06801*^*/CyO and cn*^*1*^ *P{PZ}lid*^*10424*^*/CyO; ry*^*506*^*)* have been obtained from Bloomington Drosophila Stock Center. Meiotic recombination checkpoint suppression was obtained by a heterozygous mutation, *mnk*^p6^ (*DmChk2*; [73]). A DSB repair mutation, *spnA*^*1*^ [74], was used as control. Flies carrying both *spnA*^*1*^ and *mnk*^*p6*^ mutations as well as flies carrying *mnk*^*p6*^, *Gal4*::*VP16-nos*.*UTR* and RNAi construct were obtained by standard successive genetic crosses. *Vilya*^*3XHA*^ transgenic flies (*w; pUASp-CG2709-HA*^*2*^*/CyO*) have been kindly provided by R.S. Hawley [37].

### Molecular techniques

Standard molecular techniques were used throughout [75]. For generating rat anti-Cid antibodies, the full-length Cid coding region was introduced into the Gateway (Invitrogen) entry vector pENTR, and then into destination vector pGEX4T1-Gateway (Amersham) for GST- fusion. GST-Cid was expressed in BL21/pLysS, and purified from the insoluble fraction after it was run on a SDS gel. For generating a rat anti-C(3)G antibody, full-length C(3)G has been fused with GST, and the protein has been expressed in BL21/pLysS and purified from a soluble fraction using glutathion beads. These purified proteins were used for generating antisera by Diagnostic Scotland. pCasper4-based plasmids carrying wild-type *lid* and catalytically inactive lid[JmjC*] driven by the native promoter [23] were kindly provided by J. Secombe, and used to generate transformants (The Best Gene). The lid[JmjC*] mutant contains changes of His637 and Glu639 to Ala [35].

Flies containing both RNAi constructs for *lid* and *lsd1* or *lid* and *p53* were identified by genotyping using wing PCR. DNA preparations from fly wings have been done according to [76].

Fluorescent *in situ* hybridisation (FISH) of ovaries was carried out as previously described [77,78]. The 359-bp repeat was obtained by PCR amplification from *Drosophila* genomic DNA, using primers designed from the published sequence [79]. An oligonucleotide corresponding to the dodeca satellite labelled at the 3’ end has been used as a pericentromere 3 FISH probe [77], while an oligonucleotide corresponding to the AACAC repeat has been used as a pericentromere 2 FISH probe.

To quantify the level of *Kdm5/lid* mRNA, RT-qPCR was carried out as previously described [80] except 7–10 pairs of ovaries from adult females (matured for 3–5 days at 25°C) were used. Three biological replicates were carried out for *Kdm5/lid* RNAi and *Kdm5/lid* mutant with or without transgenes, except for two biological replicates of *Kdm5/lid* mutant flies carrying a lid[JmjC*] transgene (**S5 Fig**). *Actin5C* was used as a control for normalization.

### Cytological techniques

Ovaries and mature oocytes were immunostained and analysed according to [81,82], respectively. C(3)G and SMC1 immunostaining was carried out as previously described [33]. Primary antibodies were used as follows: mouse monoclonal anti-Lamin antibody (1/100; ADL67.10; Developmental Studies Hybridoma Bank), rabbit anti-γH2Av antibody (1/100; [27]), rat anti-C(3)G antibody (1/100; this study), rat anti-SMC1 antibody (1/100, kindly provided by C. Sunkel; [83]), guinea pig anti-SMC1 and anti-SMC3 (1/2,000 and 1/1,000 respectively, kindly provided by S.E. Bickel; [6]), rat anti-Cid antibody (1/100; this study), rabbit anti-Cid antibody (1/800, Active Motif), rabbit anti-H3K4me3 antibody (1/100; Active Motif), rat monoclonal anti-HA antibody (1/100; 3F10; Roche), mouse monoclonal anti-Hts antibody (1/100; 1B1; Developmental Studies Hybridoma Bank) and mouse monoclonal anti-α-tubulin antibody (1/250; DM1A; Sigma). Secondary antibodies conjugated with Cy3, Alexa488 or Cy647 (Jackson Lab or Molecular Probes) were used at a 1/250 dilution. DNA was counterstained with DAPI (0.4 μg/ml; Sigma).

Immunostained samples were examined by an Axioimager (Zeiss) attached to a confocal laser scanning head, LSM5 Exciter (Zeiss). Either one Z-plane or a maximum intensity projection of selected Z-planes is shown in the figures after contrast and brightness were adjusted uniformly across the field using ImageJ (NIH).

To quantify the intensity of H3K4me3 levels, ImageJ software was used. The area and the Z-planes containing the karyosome were selected, and the sum of H3K4me3 signal intensity in the karyosome area of all selected Z projections was measured. The background level in an area in the nucleoplasm was estimated by measuring the sum signal intensity of the Z planes. The total karyosome-specific H3K4me3 signal was estimated to be the sum signals in the karyosome area subtracted by the background signal of the equivalent size. To quantify the intensity of Vilya^3XHA^ foci, the areas of Vilya^3XHA^ foci and nucloplasmic background were selected, and maximum signal intensity of the background area was subtracted from the maximum signal intensity of the foci area.

Fisher's exact test and t-test were used for categorical and parametric data, respectively.

## Supporting Information

S1 Fig Co-depletion of *lsd1* does not enhance or rescue the *Kdm5/lid* RNAi prophase I defects. (A) Normally formed karyosome and clustered centromeres in control and single *lsd1* RNAi, and abnormal karyosomes and unclustered centromeres in single *Kdm5/lid* RNAi as well as *Kdm5/lid lsd1* double RNAi. (B) Filamentous staining of the SC protein C(3)G in control and single *lsd1* RNAi, and spots of C(3)G staining in single *Kdm5/lid* RNAi and *Kdm5/lid lsd1* double RNAi at region 3/stage 2. (C) Quantification of karyosome morphology and the number of centromere clusters at stage 3–6, and the localisation pattern of C(3)G at region 3/stage 2 in *Kdm5/lid lsd1* single and double RNAi with control. *** indicates a significant difference in the pattern of distribution from the control (p<0.001). No significant differences are found between *Kdm5/lid* single RNAi and *Kdm5/lid lsd1* double RNAi (p>0.5). n≥21. (D) Closely paired signals of the pericentromeric dodeca satellite specific to chromosome 3 (periCen3) and pericentromeric 359-bp repeats specific to the X chromosome (periCenX) that co-localise with CenpA/Cid foci in both control and *lsd1* RNAi oocytes at stage 5. All stage 4–6 oocytes examined had closely paired pericentromere signals of chromosome X and 3 (n≥13). Scale bars = 5 μm.
(PDF)

S2 Fig *Kdm5/Lid* RNAi leads to defects in maintenance of centromere clustering. (A) Centromere clustering in pre-meiotic nuclei of 16-cell cysts (16 cc) in wild-type and *Kdm5/lid* RNAi ovaries. Parts of the germarium containing 16-cell cyst (circled) are shown in the left panels (Scale bar = 5 μm), and for each condition a magnified image of one cell is shown (the right panels; Scale bar = 3 μm). The anterior end of the germarium is oriented towards the top. The stage of each cyst was determined using the morphology of fusome visualised by Hts. (B) Increased number of centromere clusters in *Kdm5*/*lid* RNAi oocytes in comparison to control RNAi in region 3. The SC component C(3)G was used to identify oocytes. Scale bar = 3 μm. (C) The number of centromere clusters in pre-meiotic and meiotic nuclei at various oogenesis stages of control and the *Kdm5/lid* RNAi. cc; cell cyst, reg; region, st; stage. *** (p<0.001) indicates significant differences from control in terms of the frequency of nuclei with one or two centromere clusters. ≥32 nuclei were quantified for each pre-meiotic stage and region 2a, while ≥15 germaria were quantified for each meiotic stage. (D) Paring of homologous pericentromere region of chromosome 2 is disrupted in *Kdm5/lid* RNAi oocytes. Upper panel: closely paired signals of the pericentromeric AACAC satellite specific to chromosome 2 (periCen2) that localise closely to CenpA/Cid foci in the wild-type oocyte at stage 6, in contrast to two periCen2 foci clearly separated in the *Kdm5/lid* RNAi oocyte at stage 6. Arrows indicate Cid and periCen3 foci. Scale bar = 5 μm. Lower panel: the proportion of paired or separate pericentromeric signals (periCen2) in control and *Kdm5/lid* RNAi oocytes. Two signals separated by ≥1 μm were defined as "separate" in this quantification. ** indicates significant difference from the control (p<0.01). n≥17. (E) Closely paired signals of the pericentromeric 359-bp repeats specific to the X chromosome that co-localise with CenpA/Cid foci in the control and *Kdm5/lid* RNAi oocyte at stage 4/5. All stage 4–6 oocytes examined had closely paired signals in each RNAi condition (n≥16). Scale bar = 5 μm.
(PDF)

S3 Fig The pattern of C(3)G staining in germarium and later stages of oogenesis. (A) An overview image of C(3)G and DNA staining in a whole germarium from control and *Kdm5/lid* RNAi ovaries. The maximum intensity projection of several Z-planes is shown for each area of the germarium separated with dashed lines. The arrowheads indicate two pro-oocytes in region 2b in the control. Cysts around the region 2a/2b boundary are routinely excluded from our analysis, in order to allow confident staging in *lid* RNAi/mutant which affects SC morphology. (B) An overview image of C(3)G and DNA staining of control and *Kdm5/lid* RNAi ovaries at stage 6. Scale bars = 5 μm.
(PDF)

S4 Fig The pattern of SMC1 staining in germarium. An overview image of SMC1 and DNA staining in a whole germarium from a control and from a *Kdm5/lid* RNAi ovary. For each region of the germarium, separated with dashed lines, the maximum intensity projection of several Z-planes is shown. Scale bar = 5 μm.
(PDF)

S5 Fig *Kdm5/lid* mRNA is decreased in ovaries expressing shRNA against *Kdm5/lid* or in *Kdm5/lid* mutant ovaries. The level of *Kdm5/lid* mRNA in *Kdm5/lid* RNAi and *Kdm5/lid* mutant ovaries compared to control RNAi. The level of mRNA is significantly decreased in ovaries expressing shRNA against *Kdm5/lid*. As shRNA is expressed only in female germline cells, not in follicle cells, and mRNA is prepared from entire ovaries including follicle cells, the *Kdm5/lid* mRNA level would be even lower in female germline cells. Ovaries from *Kdm5/lid* mutant with one copy of wild-type *Kdm5/lid* (*lid[WT]*) or demethylase inactive (lid[JmjC*]) transgene showed significantly higher level of Kdm5/Lid mRNA than *Kdm5/lid* mutant without a transgene. Quantitative RT-PCR was used for the estimation. Error bars represent standard errors of the mean derived from biological triplicates except for two biological replicates of *Kdm5/lid* mutant flies carrying a lid[JmjC*] transgene. *** indicates a significant difference of the means from controls (p<0.001).
(PDF)
