# Supplementary figures and images for: Kdm5/Lid Regulates Chromosome Architecture in Meiotic Prophase I Independently of Its Histone Demethylase Activity
Source: PLoS Genet. 2016 Aug 5;12(8):e1006241. doi: 10.1371/journal.pgen.1006241 (PMC4975413; doi:10.1371/journal.pgen.1006241)

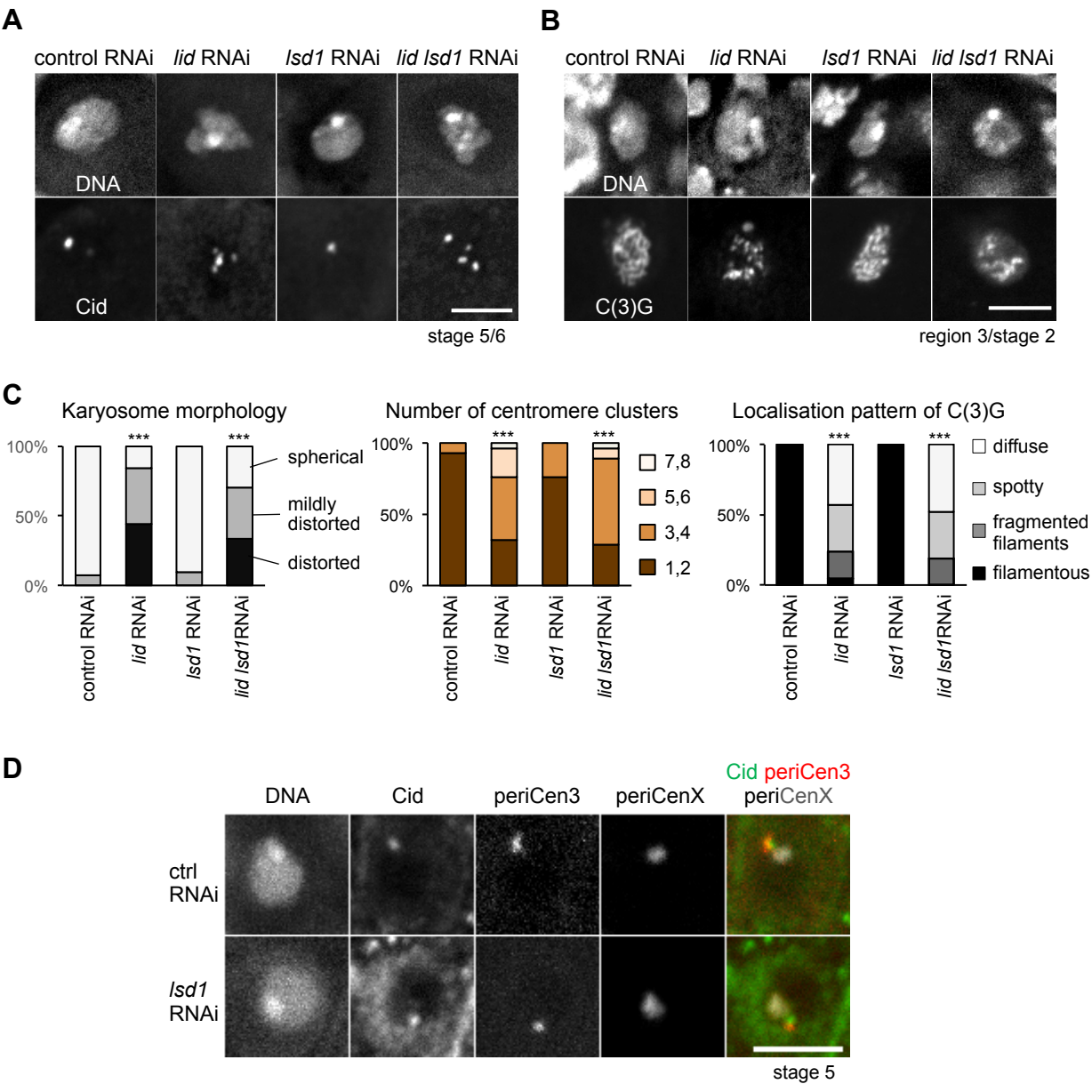

S1 Figure

Supplement: S1 Fig — (A) Normally formed karyosome and clustered centromeres in control and single lsd1 RNAi, and abnormal karyosomes and unclustered centromeres in single Kdm5/lid RNAi as well as Kdm5/lid lsd1 double RNAi. (B) Filamentous staining of the SC protein C(3)G in control and single lsd1 RNAi, and spots of C(3)G staining in single Kdm5/lid RNAi and Kdm5/lid lsd1 double RNAi at region 3/stage 2. (C) Quantification of karyosome morphology and the number of centromere clusters at stage 3–6, and the localisation pattern of C(3)G at region 3/stage 2 in Kdm5/lid lsd1 single and double RNAi with control. *** indicates a significant difference in the pattern of distribution from the control (p<0.001). No significant differences are found between Kdm5/lid single RNAi and Kdm5/lid lsd1 double RNAi (p>0.5). n≥21. (D) Closely paired signals of the pericentromeric dodeca satellite specific to chromosome 3 (periCen3) and pericentromeric 359-bp repeats specific to the X chromosome (periCenX) that co-localise with CenpA/Cid foci in both control and lsd1 RNAi oocytes at stage 5. All stage 4–6 oocytes examined had closely paired pericentromere signals of chromosome X and 3 (n≥13). Scale bars = 5 μm. (PDF) [file pgen.1006241.s001.pdf]

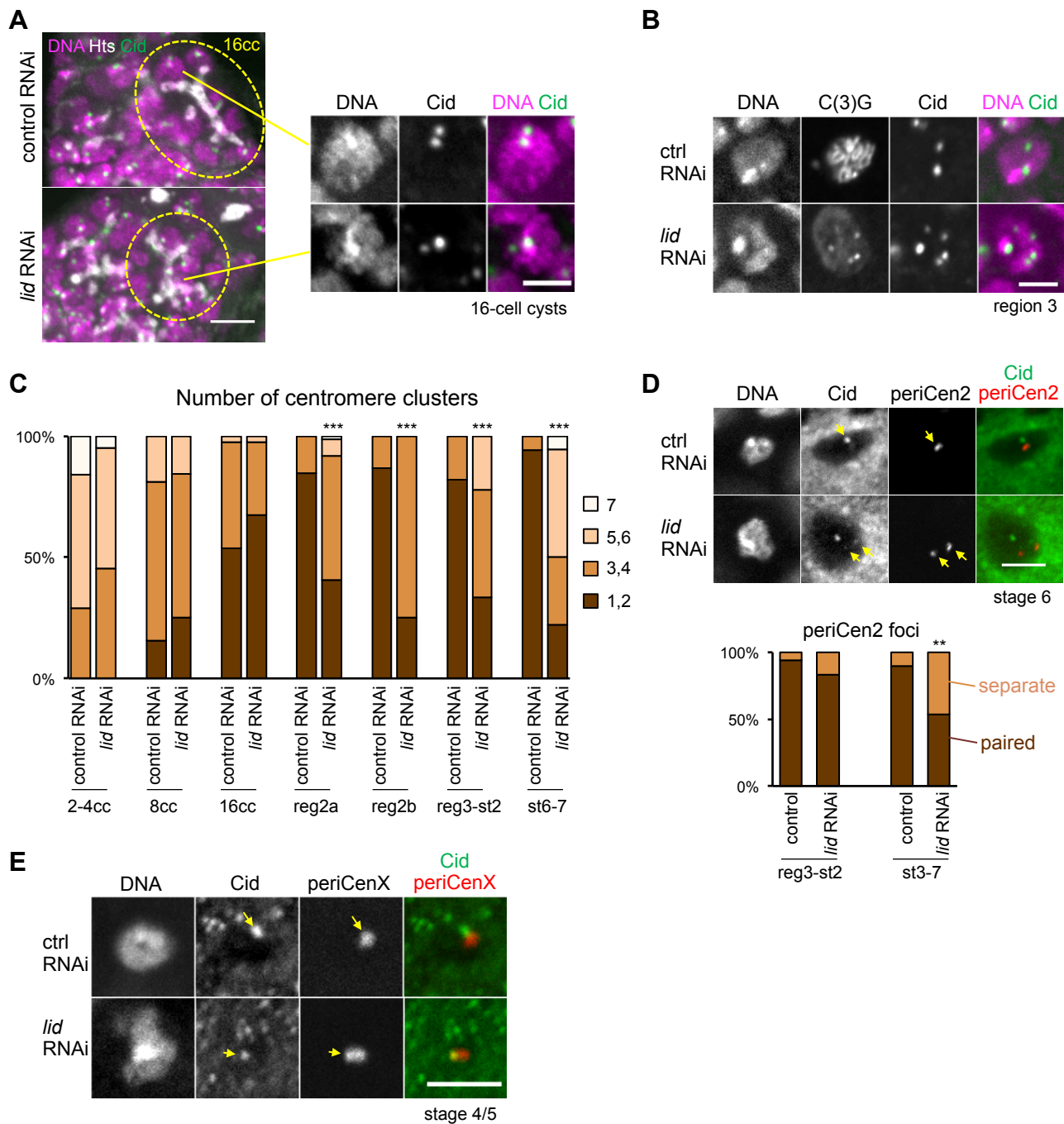

S2 Figure

Supplement: S2 Fig — (A) Centromere clustering in pre-meiotic nuclei of 16-cell cysts (16 cc) in wild-type and Kdm5/lid RNAi ovaries. Parts of the germarium containing 16-cell cyst (circled) are shown in the left panels (Scale bar = 5 μm), and for each condition a magnified image of one cell is shown (the right panels; Scale bar = 3 μm). The anterior end of the germarium is oriented towards the top. The stage of each cyst was determined using the morphology of fusome visualised by Hts. (B) Increased number of centromere clusters in Kdm5/lid RNAi oocytes in comparison to control RNAi in region 3. The SC component C(3)G was used to identify oocytes. Scale bar = 3 μm. (C) The number of centromere clusters in pre-meiotic and meiotic nuclei at various oogenesis stages of control and the Kdm5/lid RNAi. cc; cell cyst, reg; region, st; stage. *** (p<0.001) indicates significant differences from control in terms of the frequency of nuclei with one or two centromere clusters. ≥32 nuclei were quantified for each pre-meiotic stage and region 2a, while ≥15 germaria were quantified for each meiotic stage. (D) Paring of homologous pericentromere region of chromosome 2 is disrupted in Kdm5/lid RNAi oocytes. Upper panel: closely paired signals of the pericentromeric AACAC satellite specific to chromosome 2 (periCen2) that localise closely to CenpA/Cid foci in the wild-type oocyte at stage 6, in contrast to two periCen2 foci clearly separated in the Kdm5/lid RNAi oocyte at stage 6. Arrows indicate Cid and periCen3 foci. Scale bar = 5 μm. Lower panel: the proportion of paired or separate pericentromeric signals (periCen2) in control and Kdm5/lid RNAi oocytes. Two signals separated by ≥1 μm were defined as "separate" in this quantification. ** indicates significant difference from the control (p<0.01). n≥17. (E) Closely paired signals of the pericentromeric 359-bp repeats specific to the X chromosome that co-localise with CenpA/Cid foci in the control and Kdm5/lid RNAi oocyte at stage 4/5. All stage 4–6 oo [file pgen.1006241.s002.pdf]

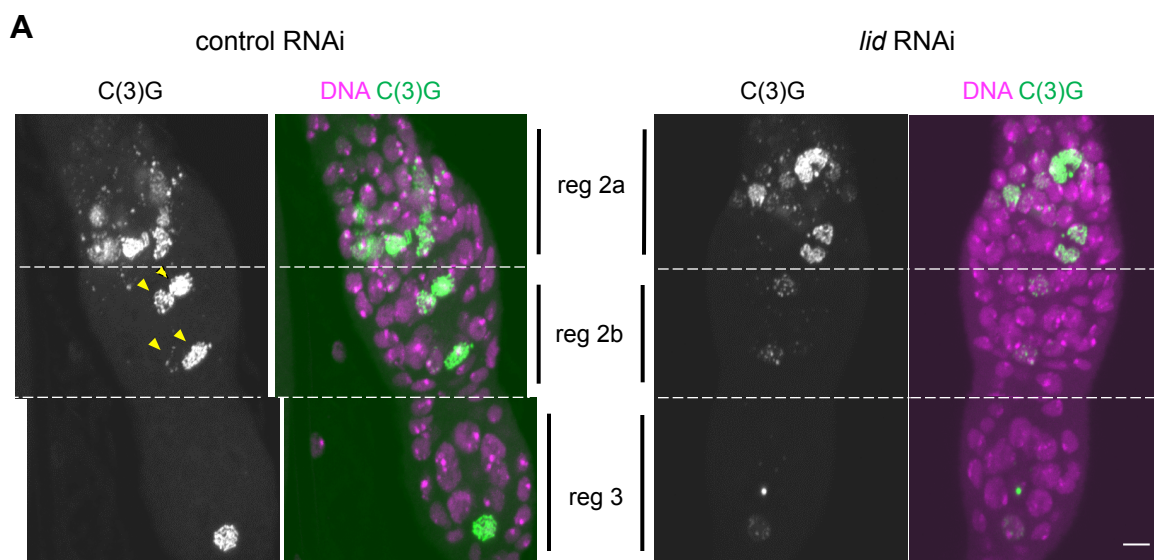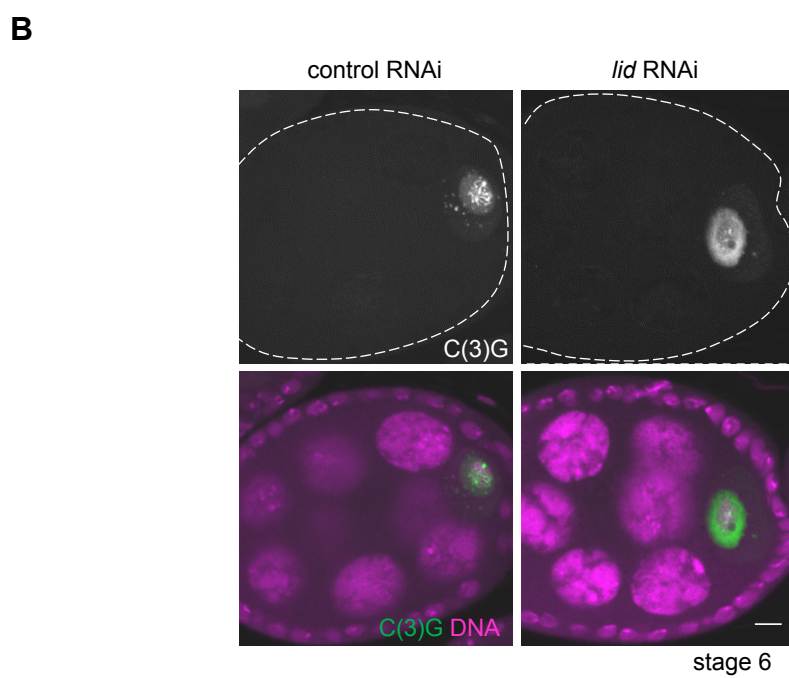

S3 Figure

Supplement: S3 Fig — (A) An overview image of C(3)G and DNA staining in a whole germarium from control and Kdm5/lid RNAi ovaries. The maximum intensity projection of several Z-planes is shown for each area of the germarium separated with dashed lines. The arrowheads indicate two pro-oocytes in region 2b in the control. Cysts around the region 2a/2b boundary are routinely excluded from our analysis, in order to allow confident staging in lid RNAi/mutant which affects SC morphology. (B) An overview image of C(3)G and DNA staining of control and Kdm5/lid RNAi ovaries at stage 6. Scale bars = 5 μm. (PDF) [file pgen.1006241.s003.pdf]

control RNAi

*lid* RNAi

SMC1

SMC1 DNA

SMC1

SMC1 DNA

reg2a

reg2b

reg3

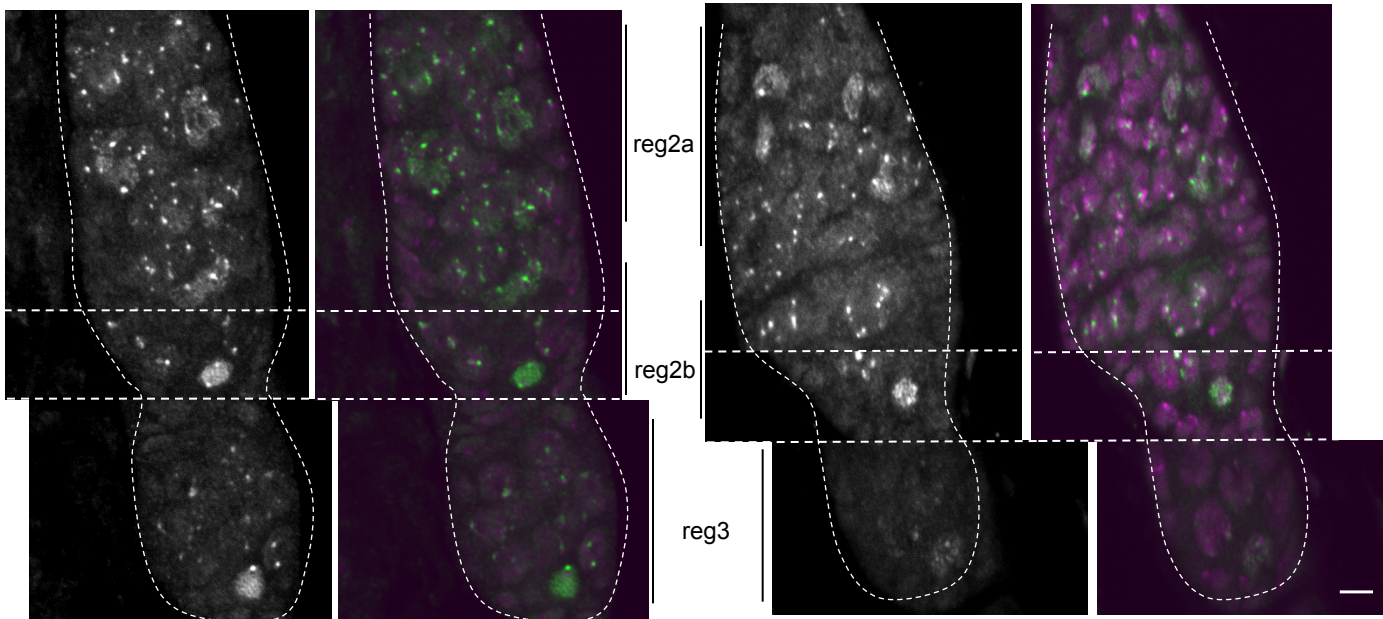

S4 Figure

Supplement: S4 Fig — An overview image of SMC1 and DNA staining in a whole germarium from a control and from a Kdm5/lid RNAi ovary. For each region of the germarium, separated with dashed lines, the maximum intensity projection of several Z-planes is shown. Scale bar = 5 μm. (PDF) [file pgen.1006241.s004.pdf]

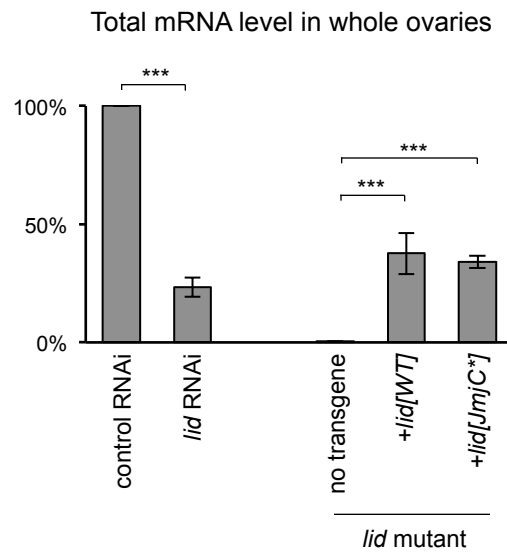

S5 Figure

Supplement: S5 Fig — The level of Kdm5/lid mRNA in Kdm5/lid RNAi and Kdm5/lid mutant ovaries compared to control RNAi. The level of mRNA is significantly decreased in ovaries expressing shRNA against Kdm5/lid. As shRNA is expressed only in female germline cells, not in follicle cells, and mRNA is prepared from entire ovaries including follicle cells, the Kdm5/lid mRNA level would be even lower in female germline cells. Ovaries from Kdm5/lid mutant with one copy of wild-type Kdm5/lid (lid[WT]) or demethylase inactive (lid[JmjC*]) transgene showed significantly higher level of Kdm5/Lid mRNA than Kdm5/lid mutant without a transgene. Quantitative RT-PCR was used for the estimation. Error bars represent standard errors of the mean derived from biological triplicates except for two biological replicates of Kdm5/lid mutant flies carrying a lid[JmjC*] transgene. *** indicates a significant difference of the means from controls (p<0.001). (PDF) [file pgen.1006241.s005.pdf]
